# Facile Synthesis of Sustainable Activated Biochars with Different Pore Structures as Efficient Additive-Carbon-Free Anodes for Lithium- and Sodium-Ion Batteries

**DOI:** 10.1021/acsomega.2c06054

**Published:** 2022-11-08

**Authors:** Glaydson Simões dos Reis, Chandrasekar Mayandi Subramaniyam, Angélica Duarte Cárdenas, Sylvia H. Larsson, Mikael Thyrel, Ulla Lassi, Flaviano García-Alvarado

**Affiliations:** † Biomass Technology Centre, Department of Forest Biomaterials and Technology, Swedish University of Agricultural Sciences, SE-901 83Umeå, Sweden; ‡ Chemistry and Biochemistry Dpto., Facultad de Farmacia, Universidad San Pablo-CEU, CEU Universities, Urbanización Montepríncipe, 28668Boadilla del Monte, Madrid, Spain; § Research Unit of Sustainable Chemistry, University of Oulu, P.O. Box 3000, FI-90014Oulu, Finland; ∥ Unit of Applied Chemistry, University of Jyvaskyla, Kokkola University Consortium Chydenius, Talonpojankatu 2B, FI-67100Kokkola, Finland

## Abstract

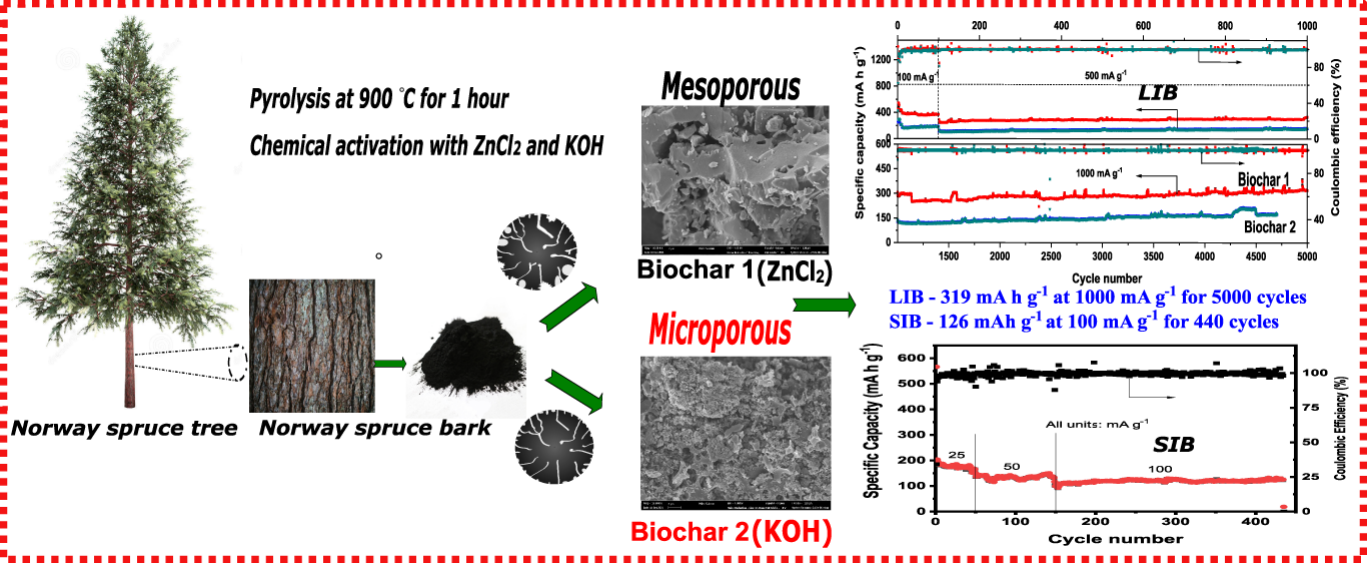

The present work elucidates facile one-pot synthesis from biomass forestry waste (Norway spruce bark) and its chemical activation yielding high specific surface area (*S*_BET_) biochars as efficient lithium- and sodium-ion storage anodes. The chemically activated biochar using ZnCl_2_ (Biochar-1) produced a highly mesoporous carbon containing 96.1% mesopores in its structure as compared to only 56.1% mesoporosity from KOH-activated biochars (Biochar-2). The latter exhibited a lower degree of graphitization with disordered and defective carbon structures, while the former presented more formation of ordered graphite sheets in its structure as analyzed from Raman spectra. In addition, both biochars presented a high degree of functionalities on their surfaces but Biochar-1 presented a pyridinic-nitrogen group, which helps improve its electrochemical response. When tested electrochemically, Biochar-1 showed an excellent rate capability and the longest capacity retentions of 370 mA h g^–1^ at 100 mA g^–1^ (100 cycles), 332.4 mA h g^–1^ at 500 mA g^–1^ (1000 cycles), and 319 mA h g^–1^ at 1000 mA g^–1^ after 5000 cycles, rendering as an alternative biomass anode for lithium-ion batteries (LIBs). Moreover, as a negative electrode in sodium-ion batteries, Biochar-1 delivered discharge capacities of 147.7 mA h g^–1^ at 50 mA g^–1^ (140 cycles) and 126 mA h g^–1^ at 100 mA g^–1^ after 440 cycles.

## 1 Introduction

The sharp increase in production of renewable energy not only is due to a higher awareness of fossil fuels’ climate impact but also provides social and economic benefits.^1^ However, solar and wind energy are characterized by non-continuous production. Therefore, energy storage systems with high energy and power densities are required to distribute electricity in real time according to society’s needs. Lithium-ion batteries (LIBs) are today’s dominant energy storage technology for electrifying portable electronics and helping build hybrid and plug-in electric vehicles,^2^ while sodium-ion batteries (NIBs) are one of the promising and viable alternatives that could potentially replace LIBs, because of the vast abundance of sodium resources, making sodium cheaper than Li. However, due to the lower electrochemical potential (+2.71 V) and higher mass of Na, NIBs deliver a low energy density and could find application where volume does not matter like in off-grid energy storage applications.^3^

Graphite, a critical anode material for battery manufacturing, is a finite fossil resource with a large CO_2_ footprint and is mined and processed at high costs.^4^ The graphite theoretical capacity of 372 mA h g^–1^ corresponds to one Li^+^ per six C atoms and has an acceptable cycle life but suffers low rate capability as compared to the lithium storage capability of porous hard carbons.^2,4,5^ Unlike in LIBs, graphite exhibits low efficiency in NIB application because Na^+^ hardly forms staged graphite intercalation compounds. Therefore, it is important to study new ways of replacing graphite for more sustainable approaches and better rate capability. This has trigged research on other types of carbon-based material anodes such as hard carbons from biomass precursors that, due to their larger interlayer carbon spacing, can be extremely effective in NIB application.^3^

Extensive research is conducted to develop innovative concepts where various biomass side-streams are employed as precursors for carbon-based materials as electrodes for LIBs and NIBs.^4,5^ Carbon materials, such as biochars, can be made from any kind of biomass, and they have good and adaptable structural and functional properties, which make them suitable to be used as anodes for both LIBs and NIBs.^3,6−10^ In addition, nowadays, battery anodes are mainly produced from fossil carbon sources or expensive materials (such as graphene and carbon nanotubes) and using biomass residues to produce efficient anodes would be both environmentally benign and economically advantageous.^2,11^ Owing to its sustainable, environmentally friendly, and cost-effective characteristics, the application of biomass opens up new possibilities for the production of green, low-cost, and high-capacity battery systems.

There are many previous studies on biochars from various resources as anodes for LIBs and NIBs. Both the biomass source and biochar production determine textural and electrochemical properties. For instance, Yu et al.^12^ prepared biomass electrodes using corn straw as a precursor, producing biochars with surface areas of up to 400 m^2^ g^–1^ and pore structures that provided active sites and a cross-linked structure shortening the distance between the Li ion and the electron and, thus, improving the specific capacity of LIBs. Battery tests showed cycling stability and a notable specific discharge capacity of 577 mA h g^–1^ after 100 cycles at 74.4 mA g^–1^. Guan et al.^13^ used hemp stems to make nanostructured porous biochars under different pyrolysis temperatures (300–800 °C). The produced biochars displayed surface areas of up to 590 m^2^ g^–1^ (with microporous and mesoporous structures) with abundance of active sites, which facilitated the cycling insertion and extraction of lithium ions, resulting in a reversible capacity of 495 mA h g^–1^ for 100 cycles. For NIBs, Zhu et al.^14^ synthesized carbon anodes from corn straw biomass at different temperatures (1200, 1400, and 1600 °C) for 2 h. The most efficient biochar anode was the one carbonized at 1400 °C. It delivered interesting electrochemical metrics: a reversible capacity of 310 mA h g^–1^ and cyclic stability at 79% capacity retention for 700 cycles.

Based on its production conditions and chemical activation method,^15^ biochar can be tailored to obtain properties that provide high-performance battery anodes.^3,7−14^ For instance, in the chemical activation process, zinc chloride (ZnCl_2_) and potassium hydroxide (KOH) are the most commonly used activation chemical agents to yield biochars with different physicochemical properties.^8−14^ Shortly, ZnCl_2_ and KOH each act differently during the activation process; the activation mechanism of KOH is based on solid–solid or solid–liquid reactions involving hydroxide reduction and carbon oxidation yielding CO and H_2_ as byproducts, while the interaction mechanism with ZnCl_2_ is based on catalytic dehydration with ZnCl_2_ acting as a skeleton during carbonization, with positive effects on the pore structure and specific surface area. Therefore, ZnCl_2_ produces biochars with more developed mesoporosity, while KOH often yields biochars with more micropores in its structure.^12^

It is reported that the combination of micro-mesopores with a high surface area interconnected with macropores has highly desired properties for improving the efficient mass transfer pathway for Li^+^/Na^+^ diffusion in porous carbon anodes.^16,17^ Furthermore, a high surface area with high mesoporosity is beneficial (1) for facilitating fast ion (Li^+^/Na^+^) diffusion through the anode to access more surface-active sites for Li^+^ or Na^+^ adsorption, which maximize both the effective intercalation area and capacitive contribution; (2) for playing a crucial role in sustaining a significant capacity even at high rates; and (3) for mitigating the volume change during cycling. Thus, basic research is required to obtain carbon materials with optimized specific surface area (SSA), porosity, and pore size distribution that are adapted to the size of electrolyte-solvated ions to therefore provide high conductivity, electrochemical response, and good physicochemical stability.^10,11^

The main goal of this work is to perform one-pot synthesis of biochars from Norway spruce bark and investigate their electrochemical properties as an alternative carbon electrode for LIBs and NIBs. The biochars were prepared using two different chemical activation, namely, using KOH and ZnCl_2_. The influence of chemical activation on the structural and chemical properties of the biochar materials and the role of the surface area and pore structure on mass diffusion and that of actives sites on capacitive processes in the biochars were unveiled by determining the respective contribution of the two storage mechanisms along with the diffusion coefficient for the lithium case. Thus, the chemically activated biochars exhibited enhanced lithium storage, retaining a specific capacity of 319 mA h g^–1^ at 1000 mA g^–1^ after 5000 cycles and a high reversible sodium storage capacity of 126 mA h g^–1^ at 100 mA g^–1^ for 440 cycles with 99% retention. Thus, it can be considered an above-par biomass anode material for LIB and NIB applications.

## 2 Materials and Methods

### 2.1 Chemicals and Reagents

The Norway spruce bark was used as the carbon precursor, and it was provided by the Holmen paper and pulp industry, Sweden. The chemical agents zinc chloride (ZnCl_2_), potassium hydroxide (KOH), and hydrochloric acid (HCl) were purchased from Sigma-Aldrich.

### 2.2 Biochar Synthesis

First, the spruce bark was dried and ball milled to an average particle size of ∼500 μm using a Fritsch Pulverisette 14 ball mill. The biochars were prepared as described elsewhere:^18,19^ first, 40 g of bark was mixed with activation chemicals (KOH or ZnCl_2_) at a ratio of 1:1 (by weight) and dispersed in 30 mL of distilled water to get a homogeneous paste. The mixtures were left for 2 h at ambient temperature and then subjected to overnight oven drying at 105 °C. The dried samples were pyrolyzed at 800 °C for 2 h at a ramp of 10 °C per min under nitrogen gas flow. After being cooled to room temperature, the samples were milled and washed with 6.0 M HCl and 1.0 M HCl for ZnCl_2_ and KOH biochars, respectively, and washed several times with distilled water until the pH value of the filtrate reached a neutral value. The ZnCl_2_-activated biochar was labeled “Biochar-1” and the KOH-activated one “Biochar-2”.

### 2.3 Biochar Material Characterization

The SSA (*S*_BET_) and porosity data of the biochars were evaluated *via* nitrogen adsorption–desorption isotherms by using a Tristar 3000 apparatus (Micrometrics Instrument Corp.). The biochars were subjected to degasification at 180 °C for 3 h, and the *S*_BET_ and pore size distribution were obtained using the Brunauer–Emmett–Teller (BET) method. Mesopore surface areas were calculated using the Barrett–Joyner–Halenda (BJH) method from desorption curves, while the micropore area values were calculated using the *t*-plot method. The morphology of the biochars was determined using field emission scanning electron microscopy (FESEM; microscope model: Zeiss Gemini). To further investigate their structure, transmission electron microscopy (TEM) images were taken using a Talos L120C microscope (FEI, Eindhoven, the Netherlands) at acceleration voltages of 20–200 kV. TEM grids were covered with 5 mL of the diluted powder samples in deionized water. Raman spectra were collected using a Bruker Bravo spectrometer (Bruker, Ettlingen, Germany) connected to a docking measuring station. Shortly, 0.5 g of each biochar powder was placed in 2.5 mL glass vials and scanned in the 300–3200 cm^–1^ spectral range at a 4 cm^–1^ resolution for 256 scans. Min–max normalization over the 1000–2000 cm^–1^ region and smoothing (nine points) were done using the built-in functions of the OPUS software (version 7, Bruker Optik GmbH, Ettlingen, Germany). No baseline correction was needed. X-ray photoelectron spectroscopy (XPS) spectra of the biochars were collected using a Kratos Axis Ultra DLD electron spectrometer equipped with a monochromated Al Kα source operated at 150 W. An analyzer of 160 eV for acquiring survey spectra and another at 20 eV for individual photoelectron lines were used. The samples were gently hand-pressed using a clean Ni spatula into a powder sample holder. Due to the electrically conductive behavior of the carbonaceous material, no charge neutralization system was used. The binding energy (BE) scale was calibrated following the ASTM E 2108 and ISO 15472 standards. Processing of the spectra was accomplished with the Kratos software. A high-resolution X-ray diffractometer (model: Bruker D8) with copper K_α_ radiation (λ = 1.5418 Å) was employed for phase determination of biochars.

### 2.4 Electrochemical Measurements

The electrochemical performance of biochars were investigated using CR 2032 lithium coin cells in a half-cell configuration. An electrode slurry containing a 9:1 weight ratio of active materials to a poly(vinylidene fluoride) (PVDF) binder was well blended in *N*-methyl-2-pyrrolidone (NMP) solvent. The slurry was tape cast over carbon-coated copper foil using a doctor blade and then vacuum dried at 120 °C overnight. The electrodes were cut into circular discs of 12 mm diameter each and weighing about 0.8–1 mg cm^–2^. The half-cell-configured coin cells were assembled in an argon-filled glovebox maintained at 0.1 ppm H_2_O and 0.1 ppm O_2_. The coin cells were fabricated using biochars as the working electrodes and lithium/sodium foil as counter/reference electrodes separated by a Whatman glass microfiber separator impregnated in a few drops of 1 M lithium hexafluorophosphate in 1:1 (v/v) ethylene carbonate (EC):diethyl carbonate (DEC) for LIBs and a sodium electrolyte consisting of 1 M NaTFSI in 1:1 (v/v) EC:DEC. All cells were tested under a constant galvanostatic current density (in mA g^–1^) between 0.002 and 3 V for LIBs and 0.002 and 2 V for NIBs (unless mentioned) with an Arbin multichannel battery tester (Texas Instruments Inc., United States) and a Neware multichannel battery tester (China). Cyclic voltammograms (CVs) were obtained at 0.1 mV s^–1^, while electrochemical impedance spectra (EIS) were obtained at frequencies between 0.1 MHz and 10 mHz at a 5 mV amplitude using BioLogic VMP3 instrument (France).

## 3 Results and Discussion

### 3.1 Physicochemical Characterization of the Biochars

**Figure 1:**
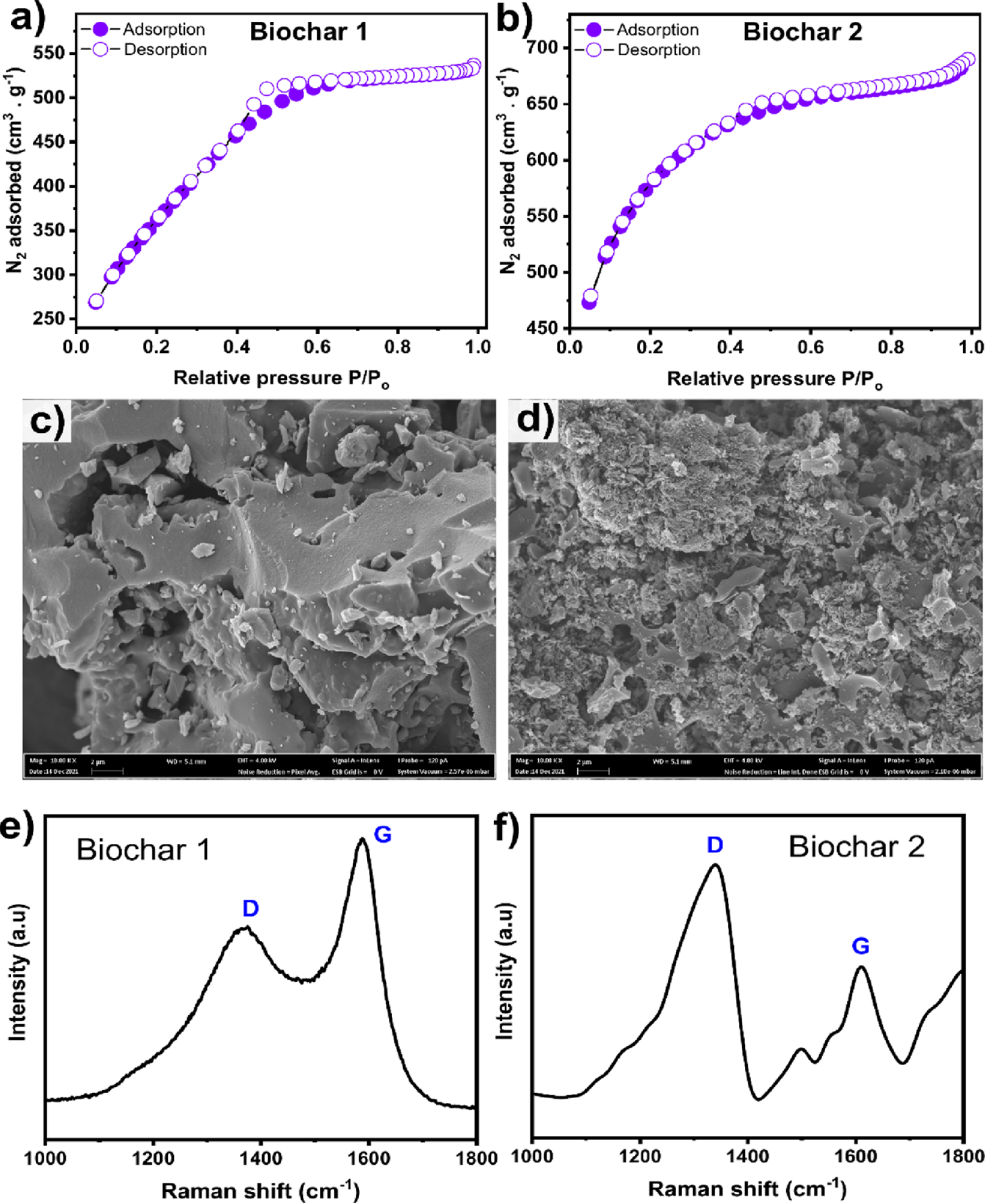
Physiochemical properties of chemically activated Biochar-1 and Biochar-2: (a,b) nitrogen adsorption–desorption isotherms; (c,d) morphological analysis using FESEM depicting the porous structures of the biochars (at 10,000× magnification); (e,f) Raman spectra showing the structural defects and degree of graphitization in the biochars.

The SSA (*S*_BET_) and pore structures of carbon materials are crucial properties for enhancing ion diffusivity by providing short distances, thereby improving their electrochemical rate performances.^6,7,17^Figure 1,ba shows the nitrogen adsorption–desorption at 77 K, which was used to determine the porosity features of the biochars. The N_2_ isotherms show that the two chemical activators’ effects on the biochars’ structural features differ, but both contain micro- and mesopores. Biochar-1 has a straight line from the initial partial pressure up to 0.4, while the isotherm of Biochar-2 displays more N_2_ adsorption at low partial pressure, which can be ascribed to the filling of micropores, suggesting the existence of more micropores. This observation is in accordance with the fact that no hysteresis exists in Biochar-2 unlike in Biochar-1, where a hysteresis exists between 0.4 and 0.6 relative pressures. This suggests that Biochar-1 prepared by chemical activation with ZnCl_2_ possesses mesopores in its structure, which is in accordance with the literature.^7,20,21^

The pore size distributions derived from the BJH plots of both biochar samples are displayed in Figure 1. Chemical activation seems to affect the pore structure of the biochar samples. Biochar-1 exhibits a much higher distribution of mesopores sized between 2.36 and 4.1 nm, while the higher peak in Biochar-2 is related to micropores (at 1.7 nm) and also exhibits the presence of mesopores. According to the pore distribution curves, both samples exhibit the presence of micropores and mesopores, which is in accordance with the data presented in Table 1. The difference in the pore size distribution of biochar samples can affect the ion-storing process since bigger pores can facilitate the diffusion pathways while the high presence of mesopores, instead micropores, guarantee the accessibility of the ions to the internal pore structure of the biochar, which can boost the efficiency of the materials as anode for battery application.

**Table 1:** Textural Properties of the Biochars

parameters (units)	Biochar-1	Biochar-2
SSA (m^2^ g^–1^)	1294	1881
mesopore surface area (m^2^ g^–1^)	1244	1055
mesopore surface area (%)	96.1	56.1
micropore area (m^2^ g^–1^)	49	825
micropore area (%)	3.9	43.9
total pore volume (cm^3^ g^–1^)	0.83	1.06
average pore size (nm)	2.65	2.20
yield (%)	38.5	12.3

From the N_2_ isotherm adsorption/desorption curves, we could ascertain that both biochars contain micro and mesoporosity and that the activation process caused important differences in its pore structures. The numerical values for textural properties are shown in Table 1. Biochar-2 had a higher *S*_BET_ value (1881 m^2^ g^–1^) than Biochar-1 (1294 m^2^ g^–1^). The mesopore surface area of Biochar-1 was 1224 m^2^ g^–1^, compared to 1055 m^2^ g^–1^ for Biochar-2. Also, in percentage, the mesopore contribution was higher for Biochar-1 (96.1%) than for Biochar-2 (56.1%). The corresponding values for the micropore surface area were as follows: Biochar-1, 49 m^2^ g^–1^ (3.9%), and Biochar-2, 825 m^2^ g^–1^ (43.9%). It was previously reported that mesopores benefit the electrochemical performance by enhancing Li^+^ or Na^+^ intercalation.^16^ The large surface area and the high percentage of mesopores in Biochar-1 can effectively help the percolation of electrolyte ions into small-sized pores. That type of pore size distribution has been reported to provide a high charge storage capability even at a high current density.^17,22^

The above observations are well-supported by the morphological analysis of biochars using FESEM as shown in Figure 1c,d. The images highlight important differences related to the use of different chemical activation agents (ZnCl_2_ and KOH); for instance, in Figure 1d, Biochar-2 shows a more broken and irregular structure full of roughness with small holes. On the other hand, Biochar-1 (Figure 1c) shows what seems to be a much denser structure, with more elongated cavities and bigger holes of different sizes and shapes. SEM analysis also indicates that the activation method induced changes on the surface characteristics of the biochars. In addition, the images show a large quantity of macropores and ultra-macropores (especially in Biochar-1). Macropores are important because they serve as vectors for electrolyte passage until smaller pores are attained (in the interior of the biochars), which can facilitate permeation of the electrolyte along with the porous structure for the following step of charge accumulation into the cavities.

Further details of the biochars are explored through TEM images (see Figure S2).^23−25^Figure S2a (Biochar-1) shows a highly amorphous and homogeneous carbon structure.^24,25^Figure S2b (Biochar-2) shows a very heterogeneous structure. Tube-like structures can be observed in Biochar-2 (see the rectangles inside the figure) that could be attributed to excessive intercalation of mobile metallic K at high temperatures resulting in bending of the carbon sheets.^11^ It also comprises some amorphous parts (see the circle inside the figure) and a certain extent of agglomeration with irregular shapes (indicated by the arrows) that can be also a reflex of the impurities observed in the X-ray diffraction (XRD) pattern (Figure S3).

The yield of the biochars is tabulated in Table 1. The yields of Biochar-1 and Biochar-2 were 38.5% and 12.3%, respectively. This result suggests that a much more violent reaction between the biomass precursor and KOH took place during the pyrolysis process. This reaction takes place *via* C–O–C and C–C bond breakage and maximizes the material’s volatilization, leading to a low carbon yield.^26,27^ On the other hand, ZnCl_2_ activation results in degradation of the cellulosic material that, combined with the dehydration during carbonization, leads to charring and aromatization of the carbon matrix, which inhibits the formation of tar and reduces mass loss, providing higher yields than when using other chemical reagents.^18,27^

Raman analysis was performed to analyze the order/disorder and degrees of graphitization in the biochars.^7,29–31^Figure 1e,f shows that both samples exhibit a disorder-induced D band (around 1340 cm^–1^) and in-plane vibrational G band (around 1585 cm^–1^), corresponding to the disordered and imperfect structures of carbon materials and the vibrations of carbon atoms with an sp^2^ electronic configuration in graphite structures. Comparing the spectra of the two samples, Biochar-1 has a bigger G peak than Biochar-2, which has a bigger D peak. These results suggest that Biochar-1 has a more ordered and higher graphitic structure, while Biochar-2 has more defects. The *I*_D_/*I*_G_ ratio of Biochar-1 goes below 1 (0.98), indicating the formation of sufficiently ordered graphite sheets, while Biochar-2 has an *I*_D_/*I*_G_ ratio of 2.0. It is well known that graphite has a high conductivity degree and behaves as an efficient material when used as an anode for battery application. A low *I*_D_/*I*_G_ value suggests that the material has more perfect and orderly graphite structures with a high graphitization degree; a high *I*_D_/*I*_G_ indicates that the material has more defects in its structure.^7,31,32^

The differences in the graphitization degrees could be related to the different activation methods. It is well-known that KOH activation enhances the expansion of carbon lattices, resulting in the increase of defects, which is reflected in the abundant pores on the carbon.^33^ This is in accordance with our textural properties highlighted in Table 1. Also, ZnCl_2_ showed a less defective structure with a more cohesive morphology/structure (as observed in SEM images). The abundance in defects can result in a less graphitic structure. In addition, Song et al.^34^ reported that activation with KOH generated biochars with less graphitized structures due to the high oxygen content. This statement is consistent with our results that displayed Biochar-2 (KOH) with a much higher oxygen content compared to Biochar-1.

**Figure 2:**
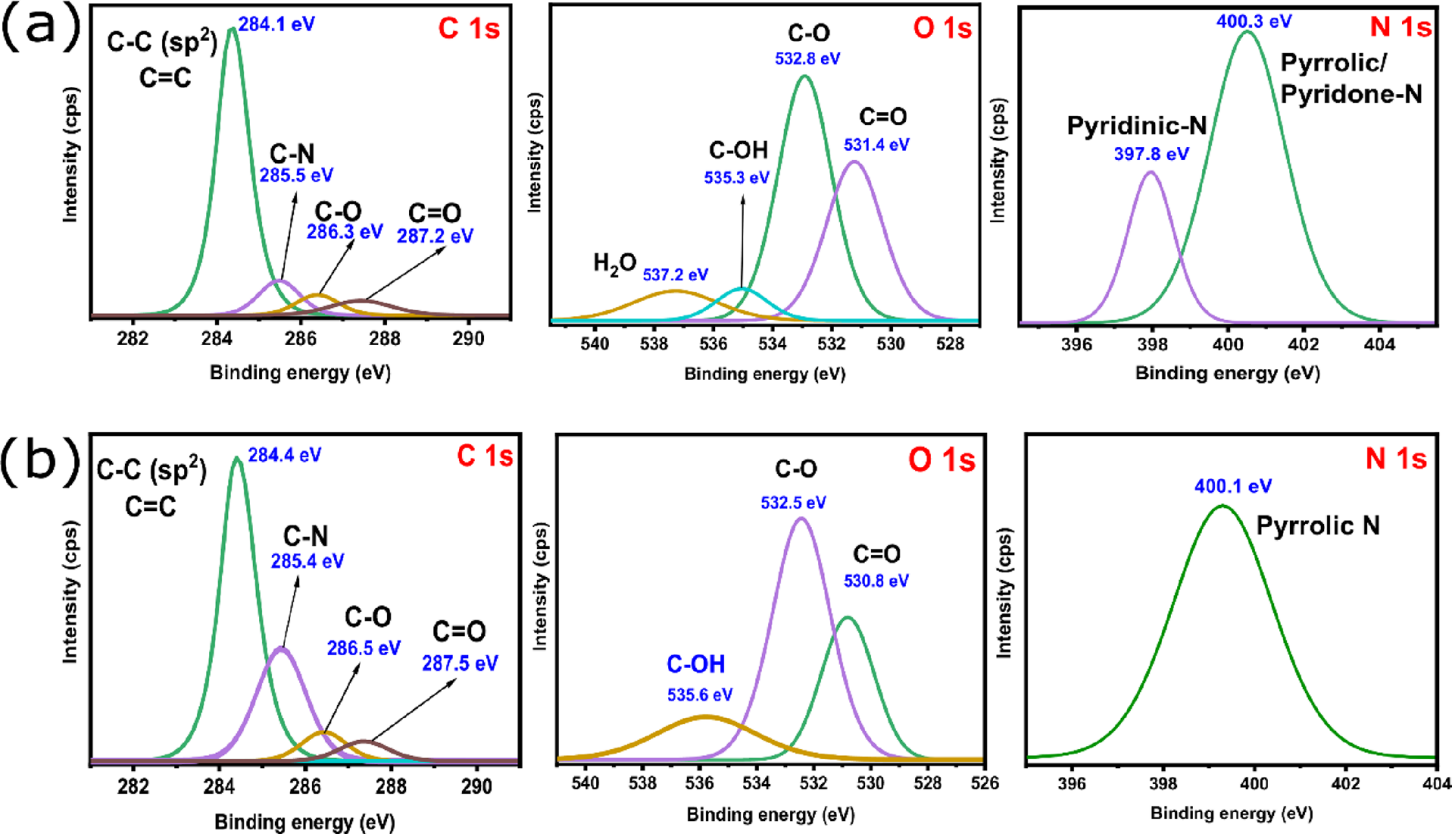
Carbon (C 1s), oxygen (O 1s), and nitrogen (N 1s) XPS spectra of (a) Biochar-1 and (b) Biochar-2.

XPS analysis was carried out to evaluate the effect of the chemical activation methods (KOH and ZnCl_2_) on the biochars’ surface element composition, chemical state, and functional groups. Figure 2 shows the XPS spectra for the carbon, oxygen, and nitrogen elements of Biochar-1 (Figure 2a) and Biochar-2 (Figure 2b). The carbon spectra (C 1s) were deconvoluted into four peaks centered at 284.1–284.4 eV, which are related to C–C and C=C bonds; at 285.4–285.5 eV, which is attributed to C–N; at 286.3 and 286.5 eV (C–O–C); and at 287.2 and 287.5 eV, which are related to C=O.^7,28,29^ For the oxygen spectra (O 1s), some differences are observed in relation to the chemical activation process. The O 1s spectra of Biochar-1 were deconvoluted into four oxygen chemical states with binding energies at around 531.4, 532.8, 535.3, and 537.2 eV, while those of Biochar-2 were deconvoluted into three peaks, 530.8, 532.5, and 535.6 eV.^7,28,29^ The peaks at 531.2 and 530.8 eV are attributed to oxygen double-bonded with carbon in carbonyl and quinone-like structures; those at 532.5 and 532.8 eV are related to oxygen singly bonded to carbon in aromatic rings, phenols, and ethers, and those at 535.1 and 535.6 eV are related to hydroxyl groups. A different peak at 537.2 eV is found only in Biochar-1, which is related to the adsorbed H_2_O–OH sub-monolayer in which −OH and H_2_O are hydrogen-bonded to each other. Nitrogen spectra (N 1s) are also shown in Figure 2. A solo peak is observed in Biochar-2, which is related to pyrrolic nitrogen,^7,28,30^ while for Biochar-1, two peaks are exhibited, one at 397.8 eV, which is related to pyridinic species, and one at 400.3 eV (pyrrolic nitrogen).^29,31^ The presence of pyridinic species in Biochar-1 can give it better electrochemical performances. N species act as the electron donor, which facilitates and improves the charge transfer.^30^ In addition, Hou et al.^30^ reported that pyridinic-N boosts the electrochemical performances of electrodes, which can be considered an important advantage for Biochar-1.

The presence of pyridinic-N could be related to the aromatization process of the biochars, which is maximized under ZnCl_2_ activation and not under KOH activation.^35^ ZnCl_2_ is an efficient dehydrating agent, and the dehydration process could result in the enhancement of the charring process and aromatization of the carbon skeleton.^36^ Peiris et al.^35^ prepared biochars from tea wastes and reported that the presence of aromatic rings derived from the nitration reaction allowed the fixation of nitrogen species in the biochars during thermochemical reactions. Moreover, its distinctive oxygen functionality and low aromatization degree^36,37^ could explain the fact why Biochar-1 (ZnCl_2_) exhibits a pyridinic-N specie on its surface that promotes electronic conductivity.

Table 2 presents the main compositions of the two biochars based on XPS and EDS elemental analyses.

**Table 2:** XPS Elemental Composition (at. %) and Elemental Analysis (%) of the Biochars

	XPS (at. %)				elemental analysis (%)				
sample ID	C 1s	O 1s	N 1s	C/O	C	N	H	O	ash
Biochar-1	94.7	4.9	0.7	19.3	94.5	0.5	1.0	2.8	1.2
Biochar-2	89.7	8.4	0.8	10.7	84.9	0.7	1.1	8.6	4.7

Biochar-1 presents higher carbon and lower oxygen contents compared to Biochar-2. The higher presence of oxygen can be related to the presence of large amounts of functionalities on the biochar surface. For instance, C/O ratios are presented, which indicates the formation and presence of hydroxyl groups on the biochars’ surfaces.^38^ The Biochar-2 sample had a lower C/O value (10.7) compared to Biochar-1 (19.3); this suggests that Biochar-2 is richer in oxygen functionalities compared to Biochar-1. During KOH activation, reactions between KOH, O-containing species in the biomass, and carbon fragments can form an abundance of vacancies in the biochar.^38^ The OH– from KOH rapidly enters these vacancies, forming a large amount of new O-containing groups (i.e., C–O, −OH, C–O, O–C–O, and −COOH groups), which in turn increases the amount of oxygen in Biochar-2.^38^

Biochar-1 presented lower ash contents compared to Biochar-2. Biochar-2 has more than three times ash content compared to Biochar-1. The ash contents are mostly related to calcite and quartz (electronic inert components).^39−41^ It is well known that high carbon and low ash contents positively affect the specific capacity of the electrode material. On the other hand, a high amount of ash can contribute to low Coulombic efficiency and cyclability of the electrode, especially if the ashes are electronically inert.

One possible reason why Biochar-2 has a higher ash content than Biochar-1 could be related to the leaching-out process.^8,19^ It is well known that the inorganic compounds formed during pyrolysis are leaching out during the washing procedure with HCl solution. The washing step was performed with HCl solutions at concentrations of 6.0 and 1.0 M for Biochar-1 and Biochar-2, respectively. It could be possible that 1.0 M HCl was not efficient enough to remove all inorganic and crystalline phases in the biochar while 6.0 M is strong enough to remove all inorganics.^8,18,19,21^

The above analysis is verified by XRD of biochars as shown in Figure S3. The biochar activated with ZnCl_2_ (Biochar-1) presents an amorphous structure with broad and weak signals of a typical highly disordered carbon structure, which is expected for porous carbon, while the KOH activation (Biochar-2) presented some distinct peaks pertaining to a crystalline impurity coexisting with the carbon amorphous phase. The presence of amorphous material is recommended for carbon electrodes due to its network pores and vacancies.^28^ The XRD pattern of Biochar-2 displays peaks that are related to a high concentration of calcite^42^ and some quartz while in Biochar-1 none of these peaks is observed. Once both biochars were prepared with the same precursor and pyrolysis conditions, a possible aspect that can contribute for higher crystallites in Biochar-2 could be related to the leaching-out process as earlier discussed in XPS discussion.

### 3.2 Electrochemical Characterization

#### 3.2.1 Biochar/Li^+^ Half-Cells

**Figure 3:**
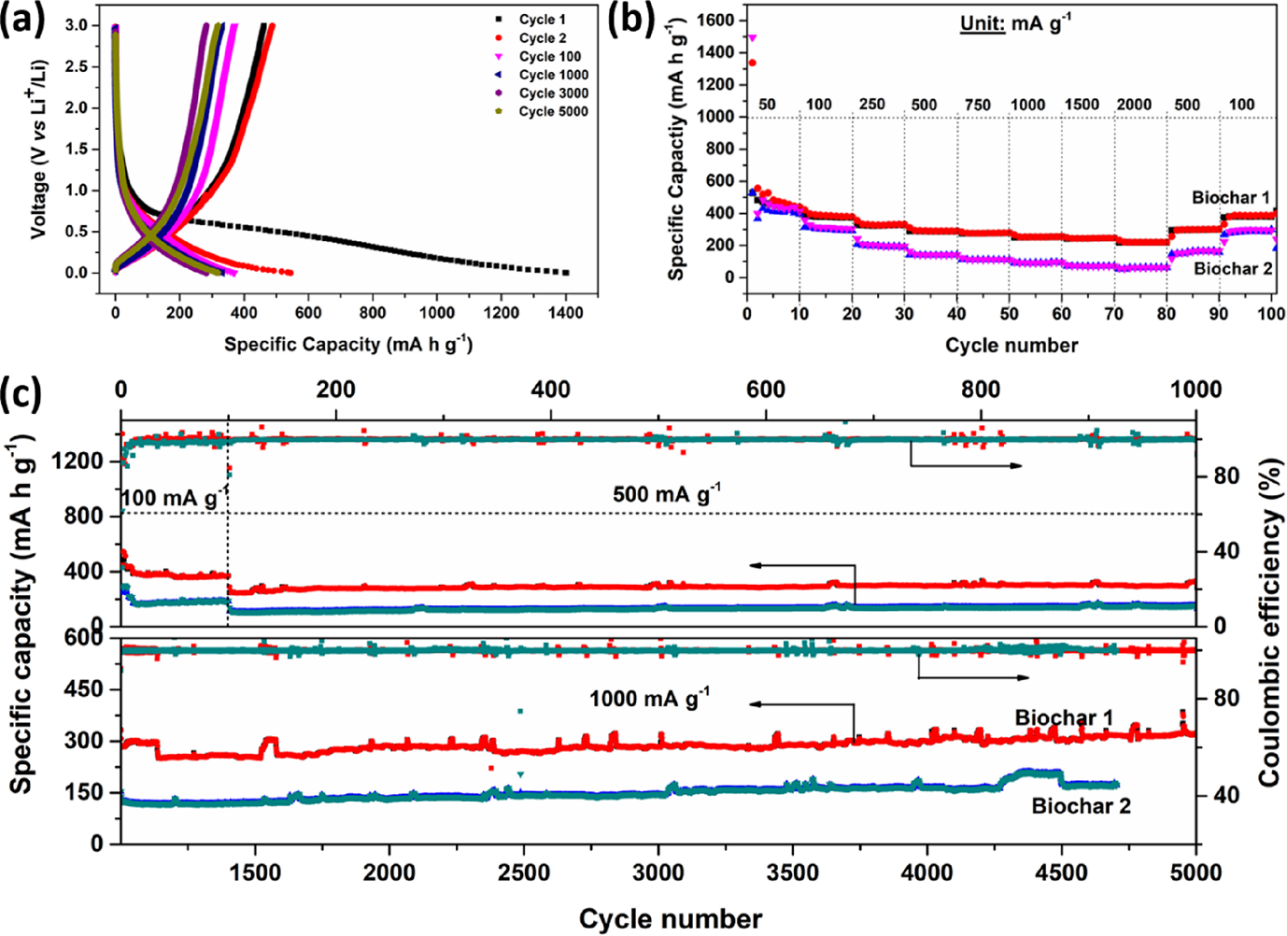
(a) Charge–discharge of Biochar-1 at various cycles, (b) rate test, and (c) long cycle stability of biochars at various current densities.

**Figure 4:**
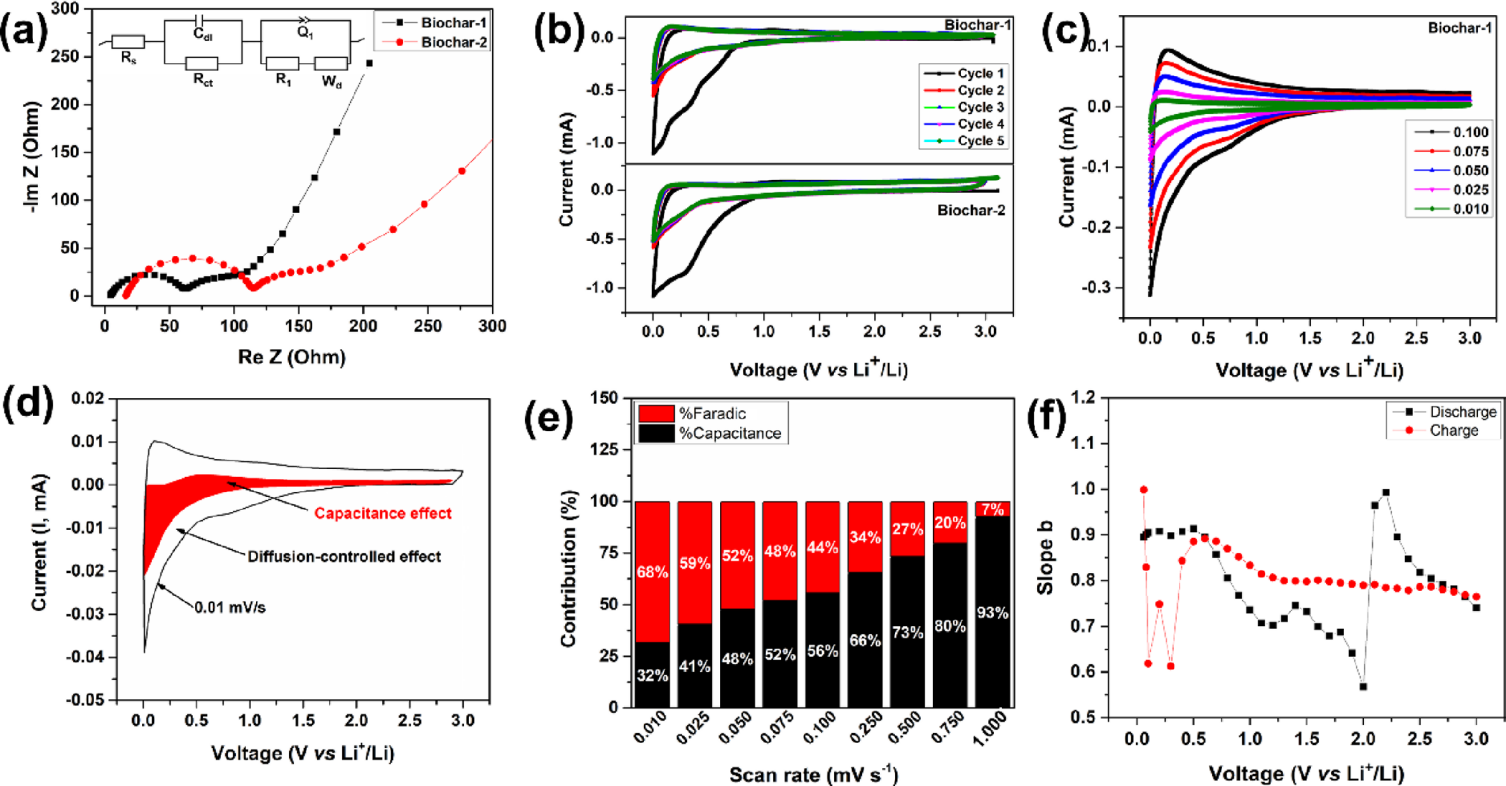
(a) Electrochemical impedance spectroscopy (EIS) of fresh cells (inset: equivalent electrical circuit); (b) cyclic voltammetry (CV) of biochars; (c) CV at different scan rates of Biochar-1; (d) CV with mapping of the diffusion-controlled mechanism and capacitive effect; (e) contribution of Faradic and capacitive effect in Biochar-1 as a function of scan rates; (f) slope *b* as a function of each potential during the charge–discharge process. From the plot, we can conclude that as the slope *b* value is greater than 0.5, the electrochemical kinetics of biochar is limited by the non-diffusive surface capacitive mechanism.

The effects of chemical treatment on the electrochemical performance of additive-carbon-free biochars were studied in a CR 2032 type coin cell in a half-cell configuration against Li metal cycled between 0.002 and 3 V. Figure 3a shows various charge–discharge plots of Biochar-1. In the first discharge cycle, the long sloping plateau below 1 V corresponds to the sum of different processes: the intercalation of Li ions into graphitic carbon, adsorption of lithium in the mesoporous carbon structures, chemical interactions of functional groups on the biochars’ surface, and formation of a solid–electrolyte interface (SEI) layer delivering the first discharge capacity of 1402.4 mA h g^–1^. Upon charging, a capacity of 461.2 mA h g^–1^ was delivered inasmuch as the two latter processes are not reversible. In the subsequent cycles, Biochar-1 delivered a reversible capacity pertaining to a Coulombic efficiency of 89.7% for the next four cycles and then improved to 99.1% for long cycles. Figure 3b shows the capacity retention capability of the active materials at different current densities to meet the demand of electric vehicles. The specific capacity of Biochar-1 decreased from 441 mA h g^–1^ at 50 mA g^–1^ to 220.2 mA h g^–1^ at 2000 mA g^–1^ and reverted to 387 mA h g^–1^ at 100 mA g^–1^ even after 100 cycles, which exhibits its excellent capacity reversibility at various current densities. In the long run, the biochars were cycled at 100 mA g^–1^ for 100 cycles followed by an increased rate of 500 mA g^–1^ for the next 1000 cycles and, finally, at 1000 mA g^–1^ for 5000 cycles (Figure 4c). Biochar-1 delivered an excellent reversible capacity of 320 mA h g^–1^ at 1000 mA g^–1^ at 99.6% Coulombic efficiency even after 5000 cycles while KOH-activated Biochar-2 could manage to deliver only 172.2 mA h g^–1^. The enhanced long cycle stability and the excellent capacity retention at a high rate of Biochar-1 could be attributed to the high conductivity due to the pyridinic-N group and the high percentage of mesopores in its carbon structures, which helps ease the transport of ions.

These characteristic features are determined by the Nyquist plot of the EIS of fresh cells (at OCV) as shown in Figure 4a. Both biochars exhibited a complete semi-circle and an incomplete depressed semi-circle followed by an inclined line pertaining to the Warburg semi-infinite diffusion characteristic pattern at high-, intermediate-, and low-frequency regions. This is represented in terms of an equivalent circuit (Figure 4a, inset) consisting of the solution resistance (*R*_s_) and the electrical double-layer capacitance (*C*_dl_) in parallel to the resistance to charge transfer (*R*_ct_) followed by a constant-phase element (*Q*_1_) and the resistance associated with the metal-ion diffusion (*W*_d_).^43^ The measured solution resistance (*R*_s_) for Biochar-1 is 4.6 Ω, while it is four-fold higher for Biochar-2. Considering the total resistance of the cells, the *R*_s_ values are insignificant and are expected to have little influence on the cell polarization difference. However, the different values of *R*_s_ can be explained as being due to the different textural properties of the biochars. At high frequencies, the intercept corresponds to the combined effects of the internal Ohmic resistances of the electrolyte and cell components such as current collectors and metallic electrical contacts.^44^ As ascertained from the cycling performance, Biochar-1 exhibited the lowest *R*_ct_ value of 57.2 Ω as compared to 97.3 Ω by Biochar-2. However, the total resistance after 5000 cycles is increased, possibly due to the contribution of the SEI resistance (Figure S4).^45^ The lithium diffusion coefficient (*D*_Li^+^_ in cm^2^ s^–1^) could be determined from the Warburg component of EIS at a low frequency using Fick’s law of diffusion as stated below:
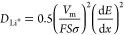
where *V*_m_ is the molar volume of carbon (cm^3^ mol^–1^), *F* is the Faraday constant (C mol^–1^), *S* is the electrode surface area (cm^2^), (d*E*/d*x*) is the first derivative of the discharge cycle, and σ is the Warburg coefficient (Ω s^–1/2^), calculated from the slope of the linear fitting *Z*′ vs ω^–0.5^ (Figure S5). The calculated diffusion coefficient of Biochar-2 at the end of the first discharge cycle is 3.1 × 10^–17^ cm^2^ s^–1^, which is 1.5 times lower than the Biochar-1 value of 4.6 × 10^–17^ cm^2^ s^–1^. This may be due to the high percentage of mesoporous structures in the carbon layers, which helps ease mass transfer and hence is reflected in their excellent electrochemical performance at different current densities.

Figure 4b depicts the CV curves for five cycles at a 0.1 mV s^–1^ scan rate. Both samples display a typical CV curve of amorphous carbon. In the first discharge cycle, the sloping curve between 1.0 and 0.45 V pertains to the irreversible formation of an SEI layer and a reduction peak (0.45–0.16 V) corresponds to intercalation of Li ions into the carbon layers to form a reversible Li_*x*_C_6_ (*x*Li^+^ + 6C + *x*e^–^ ↔ Li_*x*_C_6_) phase or surface storage in their mesoporous structures. The corresponding oxidation peak at 0.2–0.5 is observed upon reversing the polarity corresponding to the Li^+^ deintercalation process and releasing the storage electrons to the external circuit. The SEI layer is composed of decomposed electrolyte products that traps Li^+^ and is the reason behind the huge drop in the capacity from 1402.4 to 461.2 mA h g^–1^ resulting in the low first cycle Coulombic efficiency. For the subsequent cycles, the CV curves almost overlap with each other, which suggests that the intercalation and de-intercalation and surface storage of Li ions exhibited good reversibility and excellent electrochemical stability.

An elaborate kinetic investigation was carried out to elucidate possible modes of charge storage in Biochar-1. For this purpose, a half-cell was subjected to CV measurements between 0.01 and 3 V for three cycles at each scan rate starting from high (1.0 mV s^–1^) to low rates (0.01 mV s^–1^) (Figure 4c and Figure S6). The CV curve shows similar peak positions as discussed above with broadened and compressed peaks at high and low scan rates, respectively. The total area under the CV curve corresponds to the total stored charge/capacitance, which is the sum of Faradic diffusion-controlled and non-diffusive capacitive contribution mechanisms. The latter is predominant in high surface area carbonaceous materials due to charge storage by interaction with functional groups at the surface level. These contributions could be determined from CV plots at various scan rates using a power law, *i* = *av^b^*, where *i* is the measured current (mA); *v* is the scan rate (mV s^–1^); and *a* and *b* are the variable parameters, where *b* is determined from the slope of the plot of log *i* vs log *v* wherein, if *b* = 0.5 and *b* = 1, then the kinetics of the electrochemical reaction is limited by diffusion-controlled intercalation and non-diffusive capacitive mechanisms, respectively, with either ions electrochemically adsorbed or just adsorbed ions at the surface.^46,47^ Therefore, the measured current at each potential is the sum of these two contributions represented as *I* = *a*_1_*v* + *a*_2_*v*^0.5^, where *a*_1_*v* is the current from the capacitive effect and *a*_2_*v*^0.5^ is the current from the diffusion-controlled effect. The constants *a*_1_ and *a*_2_ could be determined from the slope and intercept of *I*/*v*^0.5^ vs *v*^0.5^ plots. Figure 4d represents the contributions of capacitive (red-shaded region) and Li^+^ diffusive intercalation mechanisms. The latter redox reactions are due to diffusion-controlled electrochemical kinetics and therefore take place near the redox peak positions of CV while the remaining areas are the non-diffusion capacitive effect due to surface charge storage and double-layer effects. At high scan rates, the major contribution is due to the non-diffusion capacitive effect but transcends to diffusion-limited electrochemical kinetics at low scan rates (Figure 4e). For instance, from 0.075 to 0.01 mV s^–1^ scan rates, the charge storage component from Li^+^ diffusion kinetics increased from 48 to 68% (Figure 4e), but still, the major charge storage contribution is from capacitive effects. This observation is in accordance with slope *b* as shown in Figure 4f. This is mainly due to the requirement of more time for Li^+^ diffusion as the majority interacts with surface functional groups contributing to the surface-limited rate-controlling mechanism in Biochar-1.

The electrochemical performances of our Biochar-1 LIB anodes are above par compared with many reported ones in the literature.^10,47−54^ We emphasize that our facile chemically activated biochar outclassed in terms of high surface area (1294 and 1881 m^2^ g^–1^), delivering a high initial discharge of 1402.4 mA h g^–1^, rate capability, and long cycle stability/retention of 5000 cycles even at a high current density of 1000 mA g^–1^, as a yardstick against other state-of-the-art electrode biomass LIB anodes. A more detailed comparison is tabulated in the Supporting Information (Table S1).

#### 3.2.2 Biochar/Na Half-Cells

**Figure 5:**
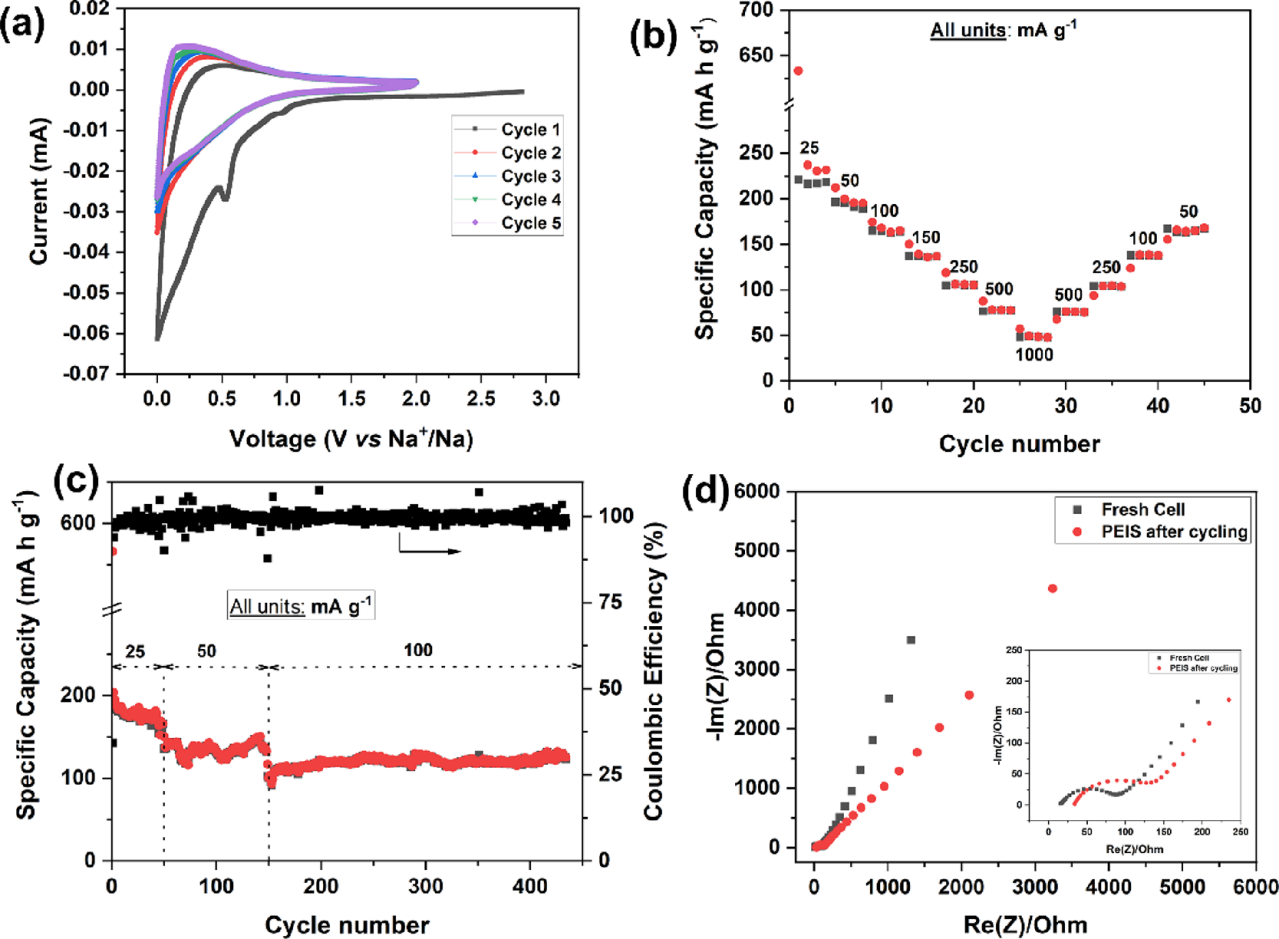
(a) Cyclic voltammetry (CV) of Biochar-1, (b) rate tests at different current densities, (c) long cycle stability of biochars at various current densities, and (d) Nyquist electrochemical impedance spectroscopy (EIS) plot of fresh cells and cells after cycling (inset: enlarged at high frequencies).

As stated earlier, the electrochemical potential of sodium (+2.71 V) is lower than that of lithium (+3.04 V) and owing to its higher atomic mass, it is anticipated that the energy density of an NIB tends to be lower than that of an LIB for the same materials of interest. However, our biochar possesses a high percentage of mesoporosity with interconnected channels for fast electron and ion transport, which could help alleviate the volume changes due to intercalation/de-intercalation of bigger Na ions and, therefore, an above-par excellent reversible cyclability is expected when this biochar is used as a sodium storing anode. In the CV plot for the first cycle, two reduction peaks at 0.53 and 0.98 V were observed. These peaks can be attributed to the decomposition of the electrolyte and formation of an irreversible SEI layer.^48^ However, these peaks disappeared in the subsequent cycles (Figure 5a), indicating that the SEI was mainly formed in the first charge/discharge.^49,50^ The CV curves from the second to the fifth cycle almost overlapped, which indicates a good reversibility for the charge storage mechanisms. The cycles overlapping after the first cycle may suggest electrolyte exposure to more active sites during sodiation/desodiation, for instance, the opening of the pores because of the material’s highly porous surface.^50^ The absence of voltage peaks in the CV profiles indicates more surface-controlled reactions during the sodiation/desodiation process.^55^

The dynamic electrochemical characteristics of Biochar-1 were evaluated by measuring the rate performance at current densities from 25 to 1000 mA g^–1^ (see Figure 5b). Biochar-1 exhibited reversible capacities of 231, 197, 167, 136, 105, and 47 mA h g^–1^ at current rates of 25, 50, 100, 200, 500, and 1000 mA g^–1^ and delivers a reversible capacity upon lowering of the current density to 50 mA g^−1^ at the end of the 45th cycle, demonstrating an excellent rate capability performance under different current densities. To further evaluate the cycling stability of Biochar-1 as a sodium anode, current densities of 25, 50, and 100 mA g^–1^ were applied to evaluate longer cycle stabilities. As can be seen in Figure 5c, the Biochar-1 anode delivered a first discharge capacity of 566.2 at 50 mA g^–1^ and 147.7 mA h g^–1^ after 140 cycles and a high reversible capacity of 126 mA h g^–1^ at 100 mA g^–1^ for 440 cycles, retaining the capacity at 99%, exhibiting excellent cycle stability. This could be attributed to the low *R*_ct_ of 94.42 Ω (from the EIS Nyquist plot, Figure 5d) maintained and the high percentage of mesoporosity that helps fast ion transport and buffer volume change during the Na^+^-intercalation and de-intercalation process. Compared with previous reports (Table S2), Biochar-1 exhibited superior Na^+^ storage performance, a higher reversible specific capacity, and better capacity retention than reported values; however, for long-term cyclability, it needs further studies. Moreover, the Biochar-1 anode showing a high Coulombic efficiency of ∼99% after 440 cycles indicates highly reversible and stable electrochemical charge storage mechanisms in our biochar anode. Although there is mainly capacity as deduced from the *I*–*V* profile (Figure 5a), a mass diffusion-controlled mechanism seems to be also present as deduced from the spike developed at a low frequency in the EIS experiment (Figure 5d).

## 4 Conclusions

In summary, we successfully synthesized chemically activated nanoporous biochar using low-cost and sustainable Norway spruce bark as a precursor to fabricate carbon electrodes with superior Li^+^ and Na^+^ storage capability. The ZnCl_2_-activated Biochar-1 possesses excellent physicochemical properties such as a high percentage of mesopores with ordered structures as compared to the disordered KOH-activated Biochar-2 sample. Biochar-1 exhibited a superior electrochemical performance of 319 mA h g^–1^ at 1000 mA g^–1^ for 5000 cycles with 99.1% Coulombic efficiency when tested in lithium half-cells. A postmortem of the electrode’s morphology after 5000 cycles shows that there is no appreciable change in the morphology of the active materials as compared to that of the fresh electrode (Figure S7a,b). Biochar-1 delivered a reversible capacity of 126 mA h g^–1^ at 100 mA g^–1^ for 440 cycles, keeping the capacity at 99% in sodium half-cells.

Due to its facile, cheap, and environmentally benign nature, the single-step biochar preparation could be extended to large-scale production of efficient biochar electrodes with improved electrochemical properties for future advanced battery applications. However, the use of biomass-based carbon materials in full cells (Figure S7c,d) remains challenging due to the large irreversible first cycle loss associated with the formation of a thick SEI layer, which is an intrinsic property of high surface area carbonaceous materials.

## Supporting Information Available

Figure S1, pore size distributions of Biochar-1 and Biochar-2. Figure S2, TEM images of Biochar-1 (a) and Biochar-2 (b). Figure S3, XRD patterns of Biochar-1 and Biochar-2. Figure S4, impedance spectra of a Biochar-1//Li cell after 5000 cycles (inset: simulated equivalent electrical circuit). Figure S5, determination of the Warburg constant from the slopes of the plots of *Z*′ vs ω^–1/2^ of (a) Biochar-1 and (b) Biochar-2. Figure S6, CV with mapping of lithium diffusion-controlled mechanism and capacitive effect in Biochar-1 at various scan rates. Table S1, comparative electrochemical performances of Biochar-1 and biomass carbonaceous materials as LIB anodes. Table S2, comparative electrochemical performances of Biochar-1 and biomass carbonaceous materials as NIB anodes. Figure S7, morphology of the Biochar-1 electrode at 3000× magnification: (a) fresh electrode and (b) after 5000 cycles. The fresh electrode composition consisted of Biochar-1 and a PVDF binder at a 9:1 weight ratio. Electrochemical performance of full cell using Biochar-1 as the anode and LiFePO_4_ as the cathode; (c) charge–discharge plot and (d) long cycling stability at various current densities (PDF)
