# Supplementary material for: Facile Synthesis of Sustainable Activated Biochars with Different Pore Structures as Efficient Additive-Carbon-Free Anodes for Lithium- and Sodium-Ion Batteries
Source: ACS Omega. 2022 Nov 8;7(46):42570–81. doi: 10.1021/acsomega.2c06054 (PMC9686188; doi:10.1021/acsomega.2c06054)
Supplement: Supplementary file 1 — ao2c06054_si_001.pdf [file ao2c06054_si_001.pdf]

Facile Synthesis of Sustainable Activated Biochars with Different Pore Structures as Efficient Additive-Carbon-free Anodes for Lithium and Sodium ions Batteries

Glaydson Simões dos Reis[1#], Chandrasekar Mayandi Subramaniyam[2#], Angélica Duarte Cárdenas[2], Sylvia H. Larsson[1], Mikael Thyrel[1], Ulla Lassi[3,4], Flaviano García-Alvarado[2]

[1-]Department of Forest Biomaterials and Technology, Swedish University of Agricultural Sciences, Biomass Technology Centre, SE-901 83 Umeå, Sweden; Glaydson.simoes.dos.reis@slu.se; sylvia.larsson@slu.se; mikael.thyrel@slu.se

[2-]Chemistry and Biochemistry Dpto., Facultad de Farmacia, Universidad San Pablo-CEU, CEU Universities, Urbanización Montepríncipe, 28668 Boadilla del Monte, Madrid, Spain; mayandi@ceu.es; angelica.duartecardenas@ceu.es; flaga@ceu.es

[3-] Research Unit of Sustainable Chemistry, University of Oulu, P.O. Box 3000, FI-90014, Oulu, Finland

[4-] Unit of Applied Chemistry, University of Jyvaskyla, Kokkola University Consortium Chydenius, Talonpojankatu 2B, FI-67100, Kokkola, Finland; ulla.lassi@oulu.fi

[#] Glaydson Simões dos Reis and Chandrasekar M Subramaniyam contributed equally to this work.

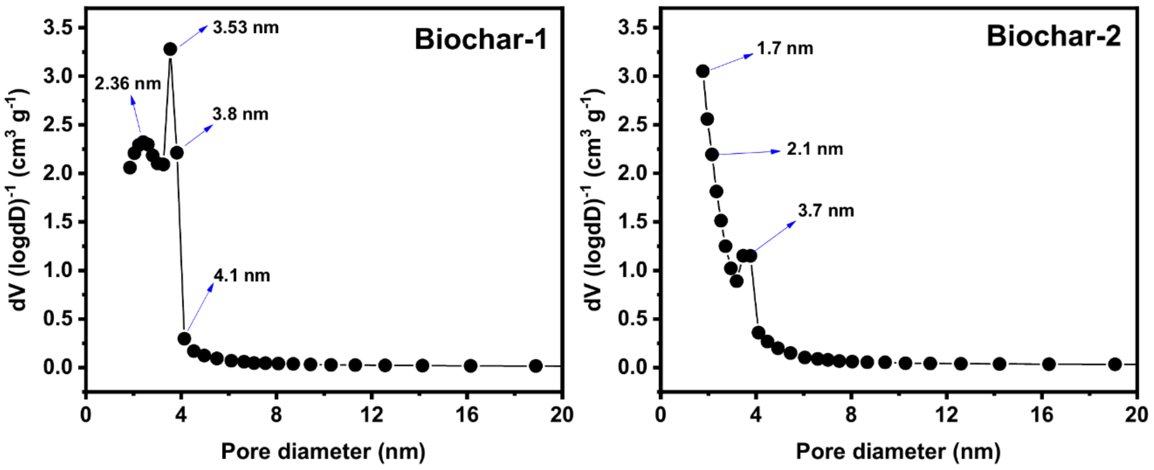

Figure S1. Pore size distribution of Biochar-1 and Biochar-2

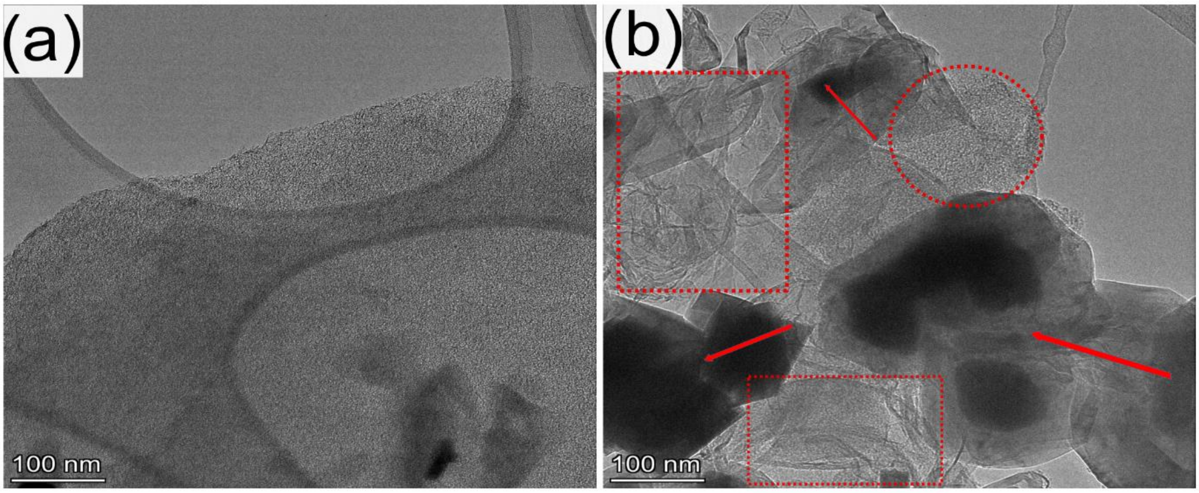

Figure S2 - TEM images of Biochar-1 (a) and Biochar-2 (b).

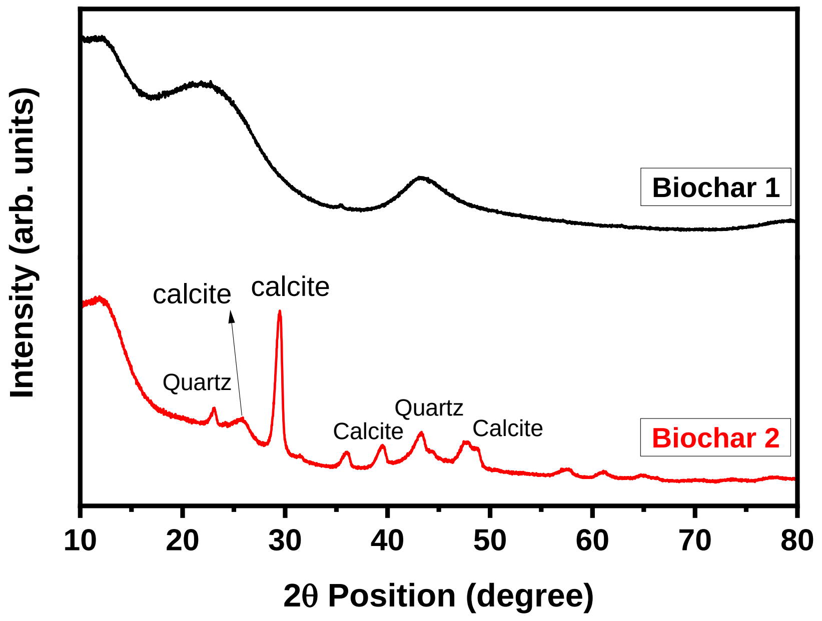

Figure S3. XRD patterns of Biochar-1 and Biochar-2

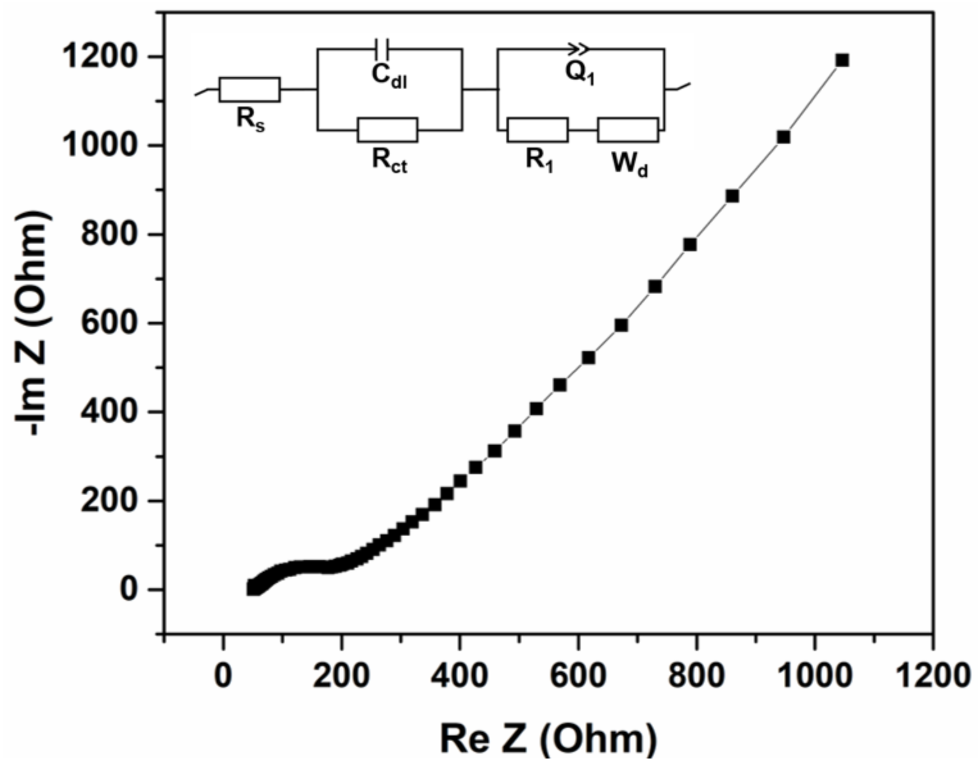

Figure S4. Impedance spectra of a biochar-1//Li cell after 5000 cycles (Insert: simulated equivalent electrical circuit).

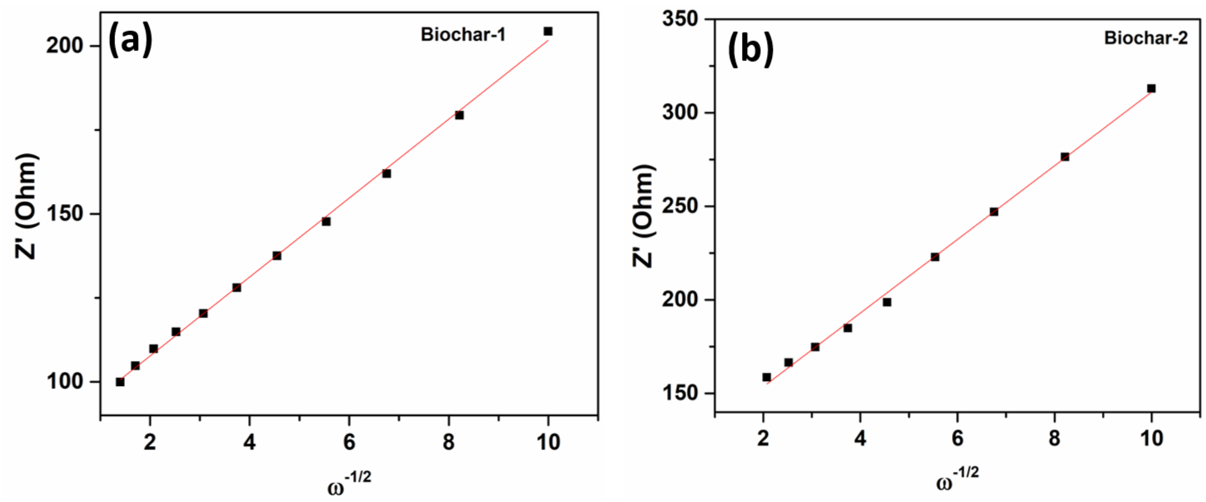

Figure S5. To determine the Warburg constant from the slope of plot Z' vs $\omega^{-1/2}$; (a) Biochar-1 and (b) Biochar-2

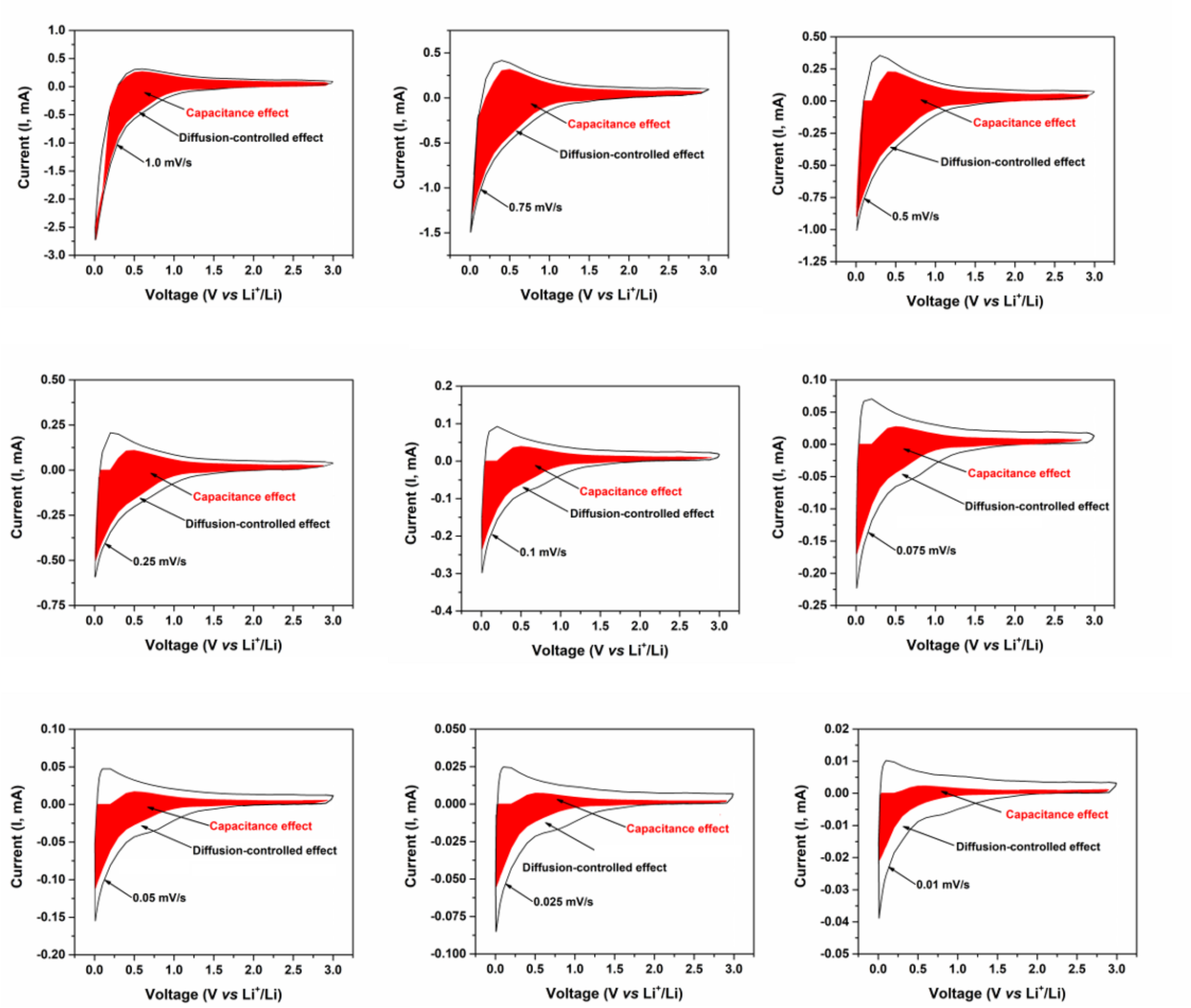

Figure S6. CV with mapping of lithium diffusion-controlled mechanism and capacitive effect in Biochar 1 at various scan rates.

Table S1. Comparative electrochemical performance of Biochar-1 with the biomass carbonaceous materials as LIB anodes.

**Biomass source**	**Synthesis method and Morphology**	**Specific surface area (SBET, m^2/g)**	**Potential (V vs Li^+/Li)**	**Current rate (mA g^{-1})**	**Initial capacity (Discharge/ Charge) (mA h g^{-1})**	**Capacity retention (mA h g^{-1})/ (cycles)**	**Rate test (mA g^{-1}), (cycle)/ capacity (mA h g^{-1})**	**Ref.**
Norwegian Spruck	***Zncl$_2$ activated mesoporous biochar***	***1294 - 1881***	***0.002 – 3.0***	***100 - 1000***	***1402.4***	369.9 - 319.4 (5000)	***2000 (10)220***	***Present work***
Wax gourd Flesh	KOH activated porous carbon with lamellar structures	834	0.01 – 1.0	37	776.6/152.2	140.9 (200)	744 (5) 76.5	(1)
Cherry Pit	Disordered carbons obtained by KOH and H_3PO_4 activation	1662	0.01 – 2.8	124	~1300/300	200 (200)	1860 (5) 70	(2)
Portobello mushroom	Carbon nanoribbon as free-standing, binder-free, and current collector-free Li-ion battery anodes	19.6	0.01 – 3.0	50	771.3/280	~260 (700)	-	(3)
Mustard seed waste	Hydrothermal synthesis of high porous spherical carbon nanostructures in-situ doped of heteroatoms (N, S)	618	0.005 – 3.0	100	~822/617	~714 (550)	500 (10) 280	(4)
Tamarind plant Seeds	Porous carbon obtained by KOH activation & annealed at 500°C for 1 h	103.51	0.01 – 2.5	200	~1037/414	~370 (100)	-	(5)
Rice Straws	High porous carbon by high temperature KOH activation method	3315	0.005 – 3.0	37.2	2041/986	-	744 (5) 257	(6)
Banana peel	High dense banana peel pseudographite by high temperature KOH activation method	217	0.001 - 2.8	100	~2150/ 1075	800 (300)	10000 (10) ~100	(7)
Jute Fiber	Micro-mesoporous carbon material using zinc chloride as an activator under high temperature carbonization in open atmosphere	1028.614	0.02 – 3.0	74.4	1173.3/ 534.1	427.2 (100)	1860 (10) 171.6	(8)

Bagasse	N,P co-doped bagasse-based sheet-like mesoporous carbon by hydrothermal activation method	1307.21 to 2118.59	0.01 – 3.0	100	2347.56/ 1186.59	816.36 (50)	2000 (200) 592.38	(9)
Coffee waste grounds	Non-porous carbonaceous materials by mechanochemical dry milling of spent coffee grounds followed by further carbonization at 800 °C.	< 10	0.0 – 3.0	100	764/~380	285 ± 5 (100)	1000 (10) 150	(10)
Spruce wood	Spruce hard carbon by pyrolysis and ball milling for size reduction	61	0.01 – 3.0	37.2	~400/ 250	300 (400)	1488 (10) ~110	(11)
Gold beard grass pollen	Mesoporous carbon powder from bee-collected pollens by pyrolysis and KOH activation	1107.447	0.01–3.0	37.2	788.99	297.283 (200) @ 2000 mA/g	5000 (-) 334.10	(12)
Avocado seeds	Non-graphitic carbonaceous anodes by pyrolysis, sulfuric acid treatment	-	0.0 – 2.0	100	~420/380	~320 (100)	400 (5) ~200	(13)
Wheat straw cellulose	Porous amorphous carbon using KOH activation agent followed pyrolysis	628	0.1 – 3.0	74.4	2750/~580	1420.5 (100)	1860 (10) 810	(14)
Coffee oil	Micrometer diameter spheroidal carbon particles by dry autoclaving method	5	0.01 – 2.0	100	~440/ 281.8	290 (200)	500 (5) ~150	(15)

Note:

1. Our works shows highest specific surface area of 1881 m^2 g^{-1} compared to others except ref.[6, 9]
2. Biochar-1 exhibited highest initial discharge capacity of 1402 mA h g^{-1} at 100 mA g^{-1} as compared to others except ref.[6,7,9,14] which are cycled at low current density.
3. The present works outclassed others in term of capacity retention of 319.4 mA h g^{-1} even after 5000 cycles at 1000 mA g^{-1} while most of the reported literatures shown cycling at low current density (20-100 mA g^{-1}) for a smaller number of cycles;
4. Our work demonstrated high-rate capability of 220 mA h g^{-1} at 2000 mA g^{-1} as compared to references[1-6, 8, 10, 11, 13, 15].

Table S2. Comparative electrochemical performance of Biochar-1 with the biomass carbonaceous materials as NIB anodes.

**Biomass source**	**Synthesis method and Morphology**	**Specific surface area (SBET, m^2/g)**	**Potential (V vs Li^+/Li)**	**Current rate (mA g^{-1})**	**Initial capacity (Discharge/ Charge) (mA h g^{-1})**	**Capacity retention (mA h g^{-1})/ (cycles)**	**Ref.**
Norwegian Spruck	***Zncl$_2$ activated mesoporous biochar***	***1294***	***0.002 – 2.0***	***25 - 100***	***~565***	***126***	***Present work***
camphor wood residues	carbonization followed by pyrolysis method, porous morphology	3.74	0.01 – 2.0	20	~324.6/ 391.8	268.1 (200)	(16)
chickpea husk	sonochemical activation method, honeycomb-like morphology	1599	0.0 – 3.0	20	~800/330	~125 (500)	(17)
Tea tomenta	High temperature treatment method, rod like morphology	13.92	0.01 – 3.0	28	~326.1	~ 262.4 (100)	(18)
Cotton roll	carbonization followed by pyrolysis method, braided fibrous morphology and hollow structure	38	0.0 – 2.0	30	~315	~ 262.4 (100)	(19)
Waste bagasse	High-temperature (1000 °C) pyrolysis. Low porosity, amorphous structure with good degree of graphitization.	92	0.01 – 3.0	25	325	60 (300)	(20)

Note:

1. Our works shows highest specific surface area of 1294 m^2 g^{-1} compared to others except ref.[17]
2. Biochar-1 exhibited highest initial discharge capacity of 565 mA h g^{-1} at 25 mA g^{-1} as compared to others except ref.[17] which are cycled at low current density.
3. The present works outclassed others in term of capacity retention of 126 mA h g^{-1} even after 440 cycles at 100 mA g^{-1} while most of the reported literatures shown cycling at low current density (20-100 mA g^{-1}) for a smaller number of cycles.

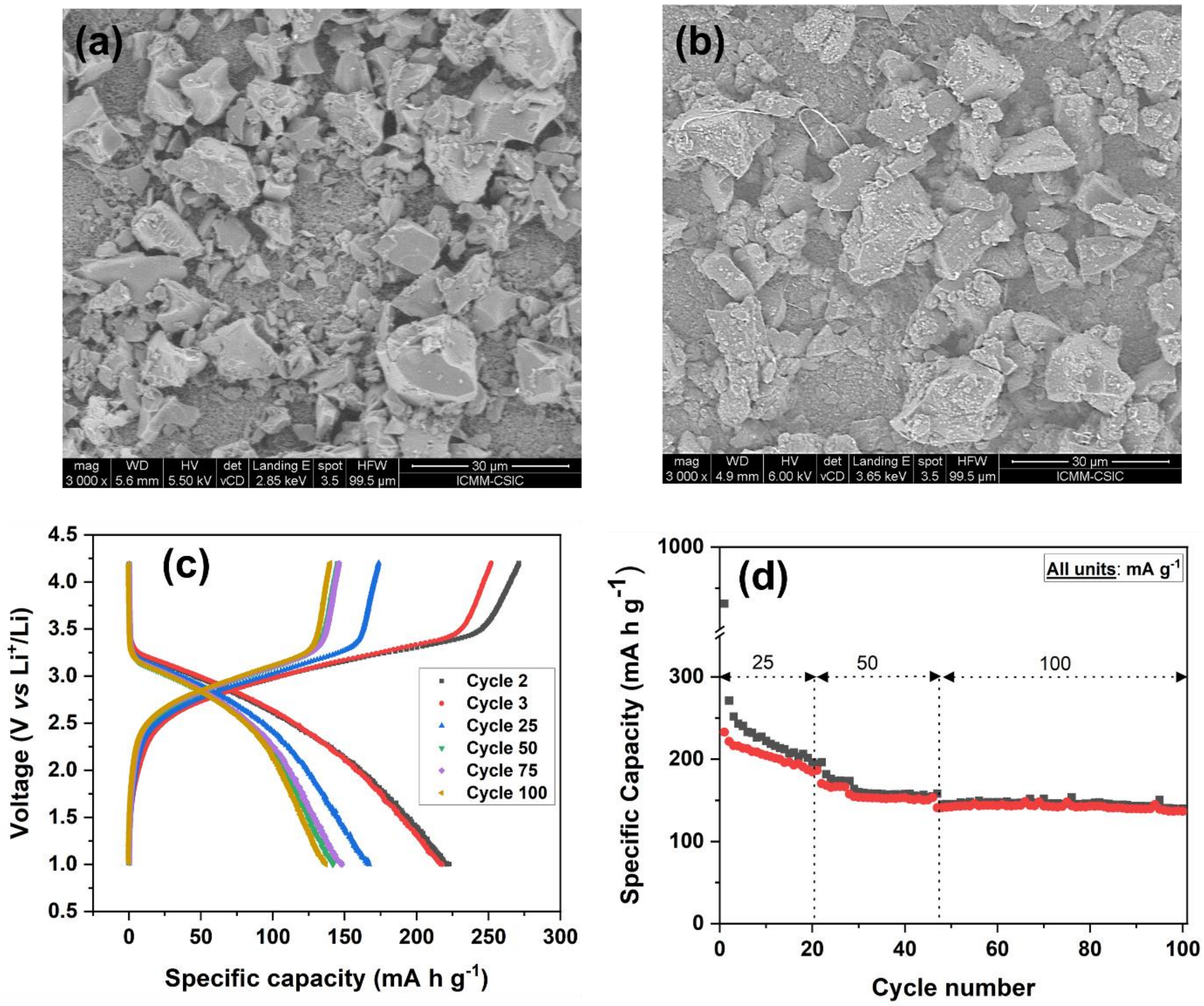

Figure S7. *Morphology of biochar-1 electrode in 3000x magnification: (a) fresh electrode and (b) after 5000 cycles. The fresh electrode composition consisted of biochar-1 and PVDF binder in the 9:1 wt. ratio, respectively; Electrochemical performance of full cell using Biochar-1 as anode and LiFePO4 as cathode; (c) charge-discharge plot and (d) long cycling stability at various current densities.*

References

(1) Zhang, Y.; Li, X.; Wang, Q.; Miao, J.; Tian, H.; Liu, X.; Shen, N.; Li, X. A wax gourd flesh-derived porous carbon activated by different activating agents as lithium ion battery anode material. *Journal of Materials Science: Materials in Electronics* **2021**, *32* (19), 23776-23785.

(2) Hernández-Rentero, C.; Marangon, V.; Olivares-Marín, M.; Gómez-Serrano, V.; Caballero, Á.; Morales, J.; Hassoun, J. Alternative lithium-ion battery using biomass-derived carbons as environmentally sustainable anode. *Journal of Colloid and Interface Science* **2020**, *573*, 396-408.

(3) Campbell, B.; Ionescu, R.; Favors, Z.; Ozkan, C. S.; Ozkan, M. Bio-Derived, Binderless, Hierarchically Porous Carbon Anodes for Li-ion Batteries. *Scientific Reports* **2015**, *5* (1), 14575.

(4) Pramanik, A.; Chattopadhyay, S.; De, G.; Mahanty, S. Efficient energy storage in mustard husk derived porous spherical carbon nanostructures. *Materials Advances* **2021**, *2* (22), 7463-7472.

(5) Panda, M. R.; Kathribail, A. R.; Modak, B.; Sau, S.; Dutta, D. P.; Mitra, S. Electrochemical properties of biomass-derived carbon and its composite along with Na2Ti3O7 as potential high-performance anodes for Na-ion and Li-ion batteries. *Electrochimica Acta* **2021**, *392*, 139026.

(6) Zhang, F.; Wang, K.-X.; Li, G.-D.; Chen, J.-S. Hierarchical porous carbon derived from rice straw for lithium ion batteries with high-rate performance. *Electrochemistry Communications* **2009**, *11* (1), 130-133.

(7) Lotfabad, E. M.; Ding, J.; Cui, K.; Kohandehghan, A.; Kalisvaart, W. P.; Hazelton, M.; Mitlin, D. High-Density Sodium and Lithium Ion Battery Anodes from Banana Peels. *ACS Nano* **2014**, *8* (7), 7115-7129.

(8) Dou, Y.; Liu, X.; Wang, X.; Yu, K.; Liang, C. Jute fiber based micro-mesoporous carbon: A biomass derived anode material with high-performance for lithium-ion batteries. *Materials Science and Engineering: B* **2021**, *265*, 115015.

(9) Zheng, S.; Luo, Y.; Zhang, K.; Liu, H.; Hu, G.; Qin, A. Nitrogen and phosphorus co-doped mesoporous carbon nanosheets derived from bagasse for lithium-ion batteries. *Materials Letters* **2021**, *290*, 129459.

(10) Luna-Lama, F.; Rodríguez-Padrón, D.; Puente-Santiago, A. R.; Muñoz-Batista, M. J.; Caballero, A.; Balu, A. M.; Romero, A. A.; Luque, R. Non-porous carbonaceous materials

derived from coffee waste grounds as highly sustainable anodes for lithium-ion batteries. *Journal of Cleaner Production* **2019**, *207*, 411-417.
(11) Drews, M.; Büttner, J.; Bauer, M.; Ahmed, J.; Sahu, R.; Scheu, C.; Vierrath, S.; Fischer, A.; Biro, D. Spruce Hard Carbon Anodes for Lithium-Ion Batteries. *ChemElectroChem* **2021**, *8* (24), 4750-4761.
(12) Kietisirirojana, N.; Tunkasiri, T.; Pengpat, K.; Khamman, O.; Intatha, U.; Eitssayeam, S. Synthesis of mesoporous carbon powder from gold beard grass pollen for use as an anode for lithium-ion batteries. *Microporous and Mesoporous Materials* **2022**, *331*, 111565.
(13) Yokokura, T. J.; Rodriguez, J. R.; Pol, V. G. Waste Biomass-Derived Carbon Anode for Enhanced Lithium Storage. *ACS Omega* **2020**, *5* (31), 19715-19720.
(14) Yu, K.; Wang, B.; Bai, P.; Liang, C.; Jin, W. Wheat Straw Cellulose Amorphous Porous Carbon Used As Anode Material for a Lithium-Ion Battery. *Journal of Electronic Materials* **2021**, *50* (11), 6438-6447.
(15) Kim, K.; Adams, R. A.; Kim, P. J.; Arora, A.; Martinez, E.; Youngblood, J. P.; Pol, V. G. Li-ion storage in an amorphous, solid, spheroidal carbon anode produced by dry-autoclaving of coffee oil. *Carbon* **2018**, *133*, 62-68.
(16) Guo, S.; Chen, Y.; Tong, L.; Cao, Y.; Jiao, H.; long, Z.; Qiu, X. Biomass hard carbon of high initial coulombic efficiency for sodium-ion batteries: Preparation and application. *Electrochimica Acta* **2022**, *410*, 140017.
(17) Ghani, U.; Iqbal, N.; Aboalhassan, A. A.; Liu, B.; Aftab, T.; Zada, I.; Ullah, F.; Gu, J.; Li, Y.; Zhu, S.; et al. One-step sonochemical fabrication of biomass-derived porous hard carbons; towards tuned-surface anodes of sodium-ion batteries. *Journal of Colloid and Interface Science* **2022**, *611*, 578-587.
(18) Wang, H.; Chen, H.; Chen, C.; Li, M.; Xie, Y.; Zhang, X.; Wu, X.; Zhang, Q.; Lu, C. Tea-derived carbon materials as anode for high-performance sodium ion batteries. *Chinese Chemical Letters* **2022**.
(19) Li, Y.; Hu, Y.-S.; Titirici, M.-M.; Chen, L.; Huang, X. Hard Carbon Microtubes Made from Renewable Cotton as High-Performance Anode Material for Sodium-Ion Batteries. *Advanced Energy Materials* **2016**, *6* (18), 1600659.
(20) Hu, H.-Y.; Xiao, Y.; Ling, W.; Wu, Y.-B.; Wang, P.; Tan, S.-J.; Xu, Y.-S.; Guo, Y.-J.; Chen, W.-P.; Tang, R.-R.; et al. A Stable Biomass-Derived Hard Carbon Anode for High-Performance Sodium-Ion Full Battery. *Energy Technology* **2021**, *9* (1), 2000730.
